# Case Report: Fatal Acute Liver Failure With Giant Cell Transformation in a Pediatric Patient Associated With MIS-C

**DOI:** 10.3389/fped.2021.780258

**Published:** 2022-01-21

**Authors:** Carolina Bonilla Gonzalez, Marcela Hincapié Echeverría, Rocio Plazas Pachón, Paola Mora Umaña, Berlly Lucia Diaz Gómez, Nathalie Gualdron Barreto

**Affiliations:** ^1^Pediatric Intensive Care Unit, Department of Pediatrics, Fundación Santafe of Bogotá, Bogotá, Colombia; ^2^Pediatric Residents Andes University, Fundación Santafe of Bogotá, Bogotá, Colombia; ^3^Department of Pathology, Fundación Santafe of Bogotá, Bogotá, Colombia

**Keywords:** liver failure, MIS-C, cytokine storm, SARS-CoV-2, case report

## Abstract

**Background:** We describe the first pediatric case of a 10-month-old boy with MIS-C who developed fulminant acute liver failure with associated giant cell transformation and a fatal outcome, after ruling out other infectious, metabolic, genetic, and autoimmune causes of liver failure following the usual algorithms for approaching the etiology. Although the patient received the main treatment strategies for liver failure, he had a fatal outcome. A clinical autopsy was considered as part of the diagnostic approach, which showed evidence of giant cell transformation.

## Introduction

Severe acute respiratory syndrome coronavirus 2019 (SARS-COV-2) is responsible for the COVID-19 pandemic. In April 2020, the UK reported several cases of hyperinflammatory processes with clinical manifestations of atypical Kawasaki disease and shock in children, suggesting a possible link to SARS-COV-2. Subsequently, the WHO and the CDC defined these cases as COVID-19-associated multisystem inflammatory syndrome in children (MIS-C), a condition presenting itself as a hyperinflammatory state leading to a cytokine storm responsible for multiorgan failure in severe presentations (1–3).

The spectrum of the disease is broad, including gastrointestinal involvement (not yet been fully elucidated), liver dysfunction, with subsequent biochemical alterations and a severe inflammatory response, and hepatic inflammation and progression to liver failure with histopathological changes of transformation to giant cells, necrosis and focal hemophagocytosis. We report a case of SARS-COV-2 infection probably presenting as rapidly progressive fulminant liver failure without respiratory symptoms (4–6).

## Case Study

A 10-month-old male infant previously healthy, with no history of hepatotoxic insults, consulted for 4 days of hypersomnia, loss of appetite, jaundice, and 24 h of dark urine and pale stools. He had a history of exposure to SARS-CoV-2 by maternal contact 45 days earlier. He was admitted stable with no stigmata of chronic liver disease.

Paraclinical tests were taken to clarify etiology by requesting hepatotropic viruses with negative results (hepatitis A,B,C, Cytomegalovirus, Epstein–Barr virus, herpes simplex virus, and human immunodeficiency virus) but with positive IgM and IgG results for SARS-CoV-2 and negative RT-PCR test for SARS-CoV-2. Results showed liver failure (Table 1). Based on the patient's laboratory results and clinical course, he met the criteria for grade I encephalopathy and acute liver failure (ALF), the reason why he was transferred to the pediatric intensive care unit (PICU).

**Table 1:** Laboratory and studies evolution.

	**Day**						
	**3**	**6**	**9**	**12**	**15**	**18**	**19**
AST	1459 U/l	873 U/l	523 U/l	382 U/l	242 U/l	132 U/l	136 U/l
ALT	2049 U/l	1129 U/l	806 U/l	498 U/l	314 U/l	181 U/l	157 U/l
TB	5.4 mg/dl	4.5 mg/dl	3.9 mg/dl	2.9 mg/dl	3.1 mg/dl	8.27 mg/dl	9.2 mg/dl
DB	3.66 mg/dl	3.1 mg/dl	2.56 mg/dl	0.74 mg/dl	1.85 mg/dl	4.94 mg/dl	5.35 mg/dl
BI	1.78 mg/dl	1.4 mg/dl	1.36 mg/dl	1.17 mg/dl	1.3 mg/dl	3.33 mg/dl	3.89 mg/dl
Albumin	3.4 g/dl	2.3 g/dl	3.6 g/dl	4.6 g/dl	4.8 g/dl	5.2 g/dl	5.2 g/dl
PT/control	21.8/10.7	17.1/11	16.0/10	17.2/10.8	17.5/10.7	16.8/11	19.3/10.9
PTT/control	41.5/25.8	39.9/26	35/26	59.1/26.0	51.8/26	40.3/26.9	121.2/26
INR	2.07	1.6	1.5	1.61	1.64	1.57	1.82
Platelets	230,000/IU	116,000/IU	70,000/lU	55,000/IU	27,000/IU	18,000/IU	26,000/IU
Ammonium	204 μg/dl	288 μg/dl	177 μg/dl	208.1 μg/dl	239.8 μg/dl	230 μg/dl	214.4 μg/dl
Fibrinogen	101.5	263.4	74	76.3	156.9	174.2	85.4
Ferritin	746.7	912	512.3	252.3	313.8	246.6	252.5
Triglycerides	–	61	–	–	58	–	–
RCP	0.681 mg/dl	0.279 mg/dl	0.289 mg/dl			1.063 mg/dl	5.23 mg/dl
PCR					0.39 μg/l	31.13 μg/l	14.57 μg/l

*Ag, antigen; Ab, antibody; HIV, human immunodeficiency virus; AST, aspartate aminotransferase; ALT, alanine aminotransferase; TB, total bilirubin; DB, direct bilirubin; PT, prothrombin time; PTT, partial prothrombin time; NV, normal value, RCP, C-reactive protein; PCR, PCT (procalcitonin)*.

Additionally, CDC criteria for COVID-19-related pediatric multisystem inflammatory syndrome (MIS-C) (2) were completed (an individual aged <21 years presenting fever (subjective fever), laboratory evidence of inflammation, as shown, evidence of clinically severe illness requiring hospitalization, with more than two-organ involvement, positive serology for SARS-CoV-2). For this reason, immunoglobulin (IVIG) and methylprednisolone were indicated; however, the patient persisted with multisystemic deterioration, initially with hepatic involvement and encephalopathy II, without deterioration of renal or cardiopulmonary function; he received management for cholestasis and hyperammonemia with hyperhydration, hepatic and neuroprotective measures, and titration of metabolic demands due to persistent hypoglycemia (7, 8).

**Figure 1:**
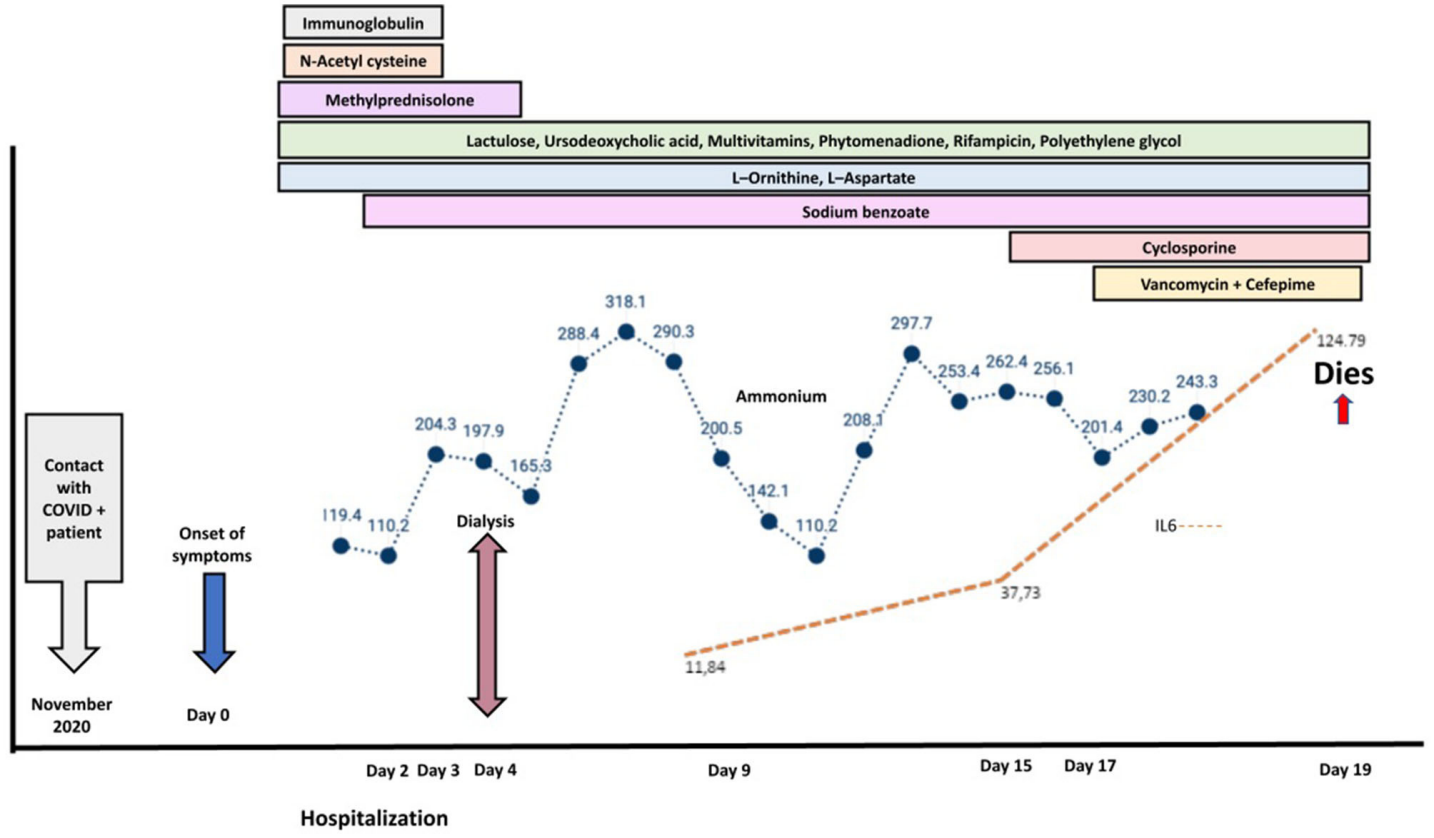
Natural history of the patient's disease. The image corresponds to the evolution of increasing ammonium (blue line) and IL-6 (orange line) values until the fatal outcome. The color bars correspond to the treatment strategies applied in the clinical course of the patient.

The patient progressed to grade IV encephalopathy (without transplant criteria), with refractory hyperammonemia, requiring hemodialysis according to the guidelines of the European Society of Gastroenterology, Hepatology, and Nutrition (ESPGHAN), with poor response and without achieving normalization of ammonium levels (Figure 1) (9). Subsequently, he developed severe and progressive coagulopathy with mucosal bleeding and brain hemorrhage which contraindicated liver biopsy. Additionally, he presented severe pancytopenia and persistent systemic involvement, with compromise of the cardiovascular (pericardial effusion), gastrointestinal (cholestasis and elevated transaminases), hematologic (coagulopathy), and neurological (encephalopathy) systems and an unmodulated inflammatory response (IR). Furthermore, he had a progressive increase of IL6 (Figure 1); therefore, cytokine storm syndrome (CS) was suspected and a bone marrow aspirate (BMA) was performed. It showed maturation and normality of hematopoietic lines with minimal histiocytes, hemophagocytosis (erythrophagocytosis), and decrease in the amount of natural killers. For this reason, immunomodulation with cyclosporine was indicated.

Given the hyperammonemia refractory to treatment, an inborn error of metabolism was suspected. Biochemical studies of small molecule diseases—quantitative amino acids in plasma and urine, qualitative short-chain organic acids in urine, and lactate–pyruvate ratio—were normal, ruling out aminoacidopathies and organic acidemias. Because of the torpid clinical evolution and persistence of abnormal clinical laboratories in a previously healthy patient, it was considered to rule out hereditary metabolic diseases by means of molecular study of critical patient—CentoICU®—with no evidence of pathogenic variants in the coding regions of the 810 genes included in the panel, related to conditions that severely affect newborns and children.

Despite sequential immunomodulation and multiple therapeutic strategies, the patient persisted with multisystem involvement associated with probable sepsis and respiratory failure secondary to alveolar hemorrhage with progression to shock refractory to vasoactive agents and died on the 17th day of hospitalization.

For the etiological approach, a clinical autopsy was performed, after obtaining parental consent, with relevant findings such as ascites (500 cc), changes due to hepatic encephalopathy, subdural hematoma in the left cerebellar hemisphere, proximal acute renal tubular necrosis, peritubular congestion, and adrenal glands with areas of hemorrhagic infiltration. External inspection of the heart showed no intracavitary thrombi. In the gastrointestinal tract, no areas of necrosis, hemorrhage, fistulas, or perforation were found. The pancreas and thyroid gland appeared normal. The hematopoietic system (spleen and lymph nodes) showed only sinusoidal congestion; no hemophagocytosis was observed in the spleen, mesenteric nodes, liver, and bone marrow (confirmed by immunohistochemistry with CD68). In the lung, a pattern of acute lung damage of diffuse alveolar type in exudative phase and subacute and acute alveolar hemorrhage (Supplementary Figure 1); hemosiderophages, focal squamous metaplasia, and occasional arteriolar thrombi; *and no acute pneumonic process* or microorganisms were observed.

The liver weighs 560 g (reference value for the age ~ 274 g); on sectioning, the cut surface is green-yellowish, without masses or other injuries. The gallbladder and bile tract are grossly unremarkable. The histological sections showed massive necrosis (90% of the tissue examined), accompanied by intracanalicular cholestasis, hepatocyte ballooning with little residual viable parenchyma in zones 2 and 3, showing hepatocytes with multinucleated giant cell transformation, mild subsinusoidal fibrosis and presence of “young” or initial collagen, and marked cholangiolar proliferation (Supplementary Figure 2). These findings raise the possibility of a case of fulminant hepatitis with giant cell transformation. Although this entity is mainly described in the neonatal period, in the literature there are also post-infantile forms in a smaller proportion, associated with infections and cholestatic conditions, and in up to 30% of cases a clear etiology cannot be identified (5, 6).

## Discussion

The SARS-CoV-2 infection associated with MIS-C has a wide spectrum of presentation in children; up to 90% may be asymptomatic, with worldwide data on hospitalization of up to 5.7% and mortality in 0.1% of cases (10). The severity in pediatrics has been related to an exaggerated immune response which starts from the second to the third week of infection, up to 79 days later, called MIS-C, with a clinical presentation similar to sepsis, Kawasaki disease, toxic shock syndrome, or macrophage activation syndrome (11). Like our report, most cases of MIS-C have positive serology for SARS-CoV-2, suggesting that it is a post-infectious condition; however, unlike our case, most of the reported cases of MIS-C have been in individuals between 8 and 10 years of age (12, 13).

In MIS-C, elevated levels of acute-phase reactants and cytopenias have been reported (2), a finding that was documented in the case presented with elevated reactants and progressive rise of IL-6 (Figure 1) correlated with clinical deterioration compatible with that described not only in MIS-C but also in a CS, without initial cytopenia (14, 15).

Given the recent description of MIS-C, information in the literature regarding the pathophysiological relationship of MIS-C and CS is limited, which makes its differentiation difficult, especially due to the undifferentiated elevation of several inflammatory biomarkers and the absence of scoring systems for its diagnosis in critically ill patients. It is, therefore, a challenge to differentiate MIS-C from other clinical spectra of CT, such as sepsis, macrophage activation syndromes, whether or not associated with an autoimmune disease, neoplasms (15–18), hyperferritinemic syndrome, or acquired hemophagocytic lymphohistiocytosis (HLH), hereditary or secondary to activation by an acute infectious condition. To date, none of the inflammatory markers can distinguish between them, and there are no pathognomonic features that define these disorders (19).

The AMO findings initially led us to suspect primary or acquired LHG syndrome, both ruled out by genetic study (molecular study of PRF1, UNC13D, STX11, or STXBP2 genes) and histopathological analysis at necropsy, with no documented evidence of hemophagocytosis in the spleen, mesenteric lymph nodes, liver, or bone marrow. The finding of non-nucleated erythrophagocytosis in AMO can also be observed in many other conditions other than HLH (14, 20). Liver involvement by SARS-CoV-2 associated with Misc following acute infection has already been described and is considered secondary to a cytopathic effect due to IR dysregulation. Chen et al. (21) reported a series of 43 pediatric cases from Wuhan, with elevated transaminase and LDH levels where only one patient had very elevated aminotransferases (ALT 7590 U/l and AST 1445 U/l), without any case of acute liver failure (ALF) or intrahepatic cholestatic pattern.

Retrospective studies of pediatric patients with MIS-C reported cases of hepatitis with increased transaminases associated with increased IR, but very low incidence of progression to liver failure (1 of 19 patients) (22), which shows that in cases of MIS-C the liver can also be one of the target organs, with an association of MIS-C and hepatitis in pediatric patients similar to the case presented.

Liver failure has been reported in adults who did not have a preexisting liver disease, but it has not been a common outcome; on the other hand, liver injury has been reported in more than 60% of patients with SARS-CoV-2 (23). There is also a case report in a 47-year-old woman who recovered from COVID-19 2 months before, with a re-positive RT-PCR test for SARS-CoV-2, who presented gastrointestinal symptoms with elevated transaminases and developed fulminant hepatic failure and died (5). Finally, we found in the literature a retrospective study of 291 patients under 21 years of age in New York where an elevation of ALT was evaluated and an elevation of median ALT above 2 times the normal value with cholestasis was described and was associated with a more severe course of disease and with a higher prevalence of multiorgan dysfunction (4).

Regarding acute hepatic failure, some hypotheses have suggested the relationship between this and COVID-19 infection. One of them proposes that CS generates liver damage by elevation of Th17, CD8, IL-2, 6, 7, and 10 lymphocytes, tumor necrosis factor-alpha, and granulocyte colony-stimulating factor 1, associated with other mechanisms of oxidative stress, hypoxic–ischemic necrosis secondary to micro- and macrovascular thrombosis, and exaggerated activity of Kupffer cells and the adrenocortical system (22, 24). A direct correlation has been suggested between the elevation of transaminases and inflammatory markers with greater severity of COVID-19 disease. Among these, IL-6 is characterized by its proliferation factor, inflammation, and mitogenic effect, in addition to hypoxia, hypoperfusion, and hypovolemia, triggering the destruction of the biliary epithelium, necrosis, and fulminant hepatitis, as well as other mechanisms of activation of secondary hemophagocytic lymphohistiocytosis (25, 26). The latter was not observed in the clinical autopsy of our patient.

Concerning SARS-CoV-2 infection associated with Misc and the histopathological finding of hepatocytes with multinucleated giant cell (GC) transformation, it has only been reported in a 35-year-old patient with SARS-COV-2 infection and biopsy with panacinar hepatitis with focal GC transformation, necrosis, and focal hemophagocytosis, with no data known to date in the pediatric population. GC transformation can usually be associated with cholestatic jaundice, hepatomegaly, and variable degrees of coagulopathy; so far, infectious agents associated with this transformation in the pediatric population include paramyxovirus, rubella, hepatitis B and C, and HIV, all of which have been ruled out in our patient. This transformation is a rare disorder of hepatocyte regeneration or degeneration in response to noxae, characterized by the presence of hepatic multinucleated cells with a broad clinical spectrum, ranging from mild acute or chronic hepatitis to the development of rapidly progressive cirrhosis and ALF (27–30).

The presence of this patient's severe liver involvement in the context of a MIS-C, probably secondary to the tropism described by SARS-COV-2, coupled with an unmodulated systemic IR syndrome that manifested as a CS, with an uncontrolled increase in cytokine production, resulting in multiorgan failure, systemic inflammation, and death (17, 27).

This is the first pediatric case to date of fulminant acute liver failure with giant cell transformation by clinical autopsy associated with MIS-C. Recognition of this phenomenon in patients with SARS-COV-2 infection remains a challenge. Data and knowledge on liver injury, diagnosis, and treatment strategies, especially targeting the MIS-C-associated cytokine storm (CS) in the context of COVID-19 infection, are lacking.

The most important limitation of our case report is that we cannot establish a causal association between MIS-C and acute liver failure; however, after a complete study, as shown, we suggest to consider MIS-C as a cause of liver failure and to use therapies that protect liver functions in patients with MIS-C which may help a better outcome of the disease. Still, there is few information about this association, making our case interesting and a possible branch of new studies (3, 31).

## Conclusions

Hepatic manifestations of SARS-COV-2 in pediatrics are usually limited to elevated transaminases, and there are few reports of AHF with fatal outcome, so the approach and management of AHF associated with this new virus challenge medical management skills. This is the first reported pediatric case of fulminant hepatitis with autopsy transformation of GC associated with SARS-COV-2.

It is necessary to study biomarkers and new therapies in the treatment, as well as a concerted therapeutic approach by a multidisciplinary team of experts, which provides access to immunomodulators (IVIG, steroids), chemotherapy (cyclosporine, cytotoxic agents), and biological agents (17, 28), which generate the blockage in the activation of cytokine signaling in this pathology.

## Data Availability Statement

The original contributions presented in the study are included in the article/Supplementary Material, further inquiries can be directed to the corresponding author/s.

## Ethics Statement

Written informed consent was obtained from the minor(s)' legal guardian for the publication of any potentially identifiable images or data included in this article.

## Author Contributions

MH and RP made the literature search and figures. BD contributed the photos and analysis of the clinical autopsy. RP, MH, CB, and PM prepared the original draft of the manuscript. RP, MH, PM, CB, NG, and BD contributed to reviewing and editing the manuscript. All authors had full access to all data in the study, participated in the interpretation, revised the manuscript, approved the final version of the manuscript for publication, and contributed important intellectual content during manuscript drafting or revision.

## Conflict of Interest

The authors declare that the research was conducted in the absence of any commercial or financial relationships that could be construed as a potential conflict of interest.

## Publisher's Note

All claims expressed in this article are solely those of the authors and do not necessarily represent those of their affiliated organizations, or those of the publisher, the editors and the reviewers. Any product that may be evaluated in this article, or claim that may be made by its manufacturer, is not guaranteed or endorsed by the publisher.

## Supplementary Material

Supplementary Figure 1 Lung. **(A)** GROSS image: on sectioning, the cut surface is reddish, firm (red hepatization—RH). No mass or gross thrombi are present. **(B)** ×4 high-power field (HPF) and **(C)** ×10 HPF—lung parenchyma with edema-e, hyaline membranes (arrow), and hemorrhage coating the alveoli (^*^).

Supplementary Figure 2 **(A)** GROSS image: The external surface is smooth brown-yellowish, with lobulations. On sectioning, the cut surface is green-yellowish, without masses or other injuries. **(B,C)** ×10 hpf—massive hepatic necrosis (Ne) (~90%), hepatocyte ballooning with little viable residual parenchyma in zones 2 and 3 (^*^), observing hepatocytes with multinucleated giant cell transformation (GC), and marked cholangiolar proliferation (cp); **(D)** ×40 hpf—hepatocytes with multinucleated giant cell transformation (arrow).
