# Supplementary figures and images for: Case Report: Fatal Acute Liver Failure With Giant Cell Transformation in a Pediatric Patient Associated With MIS-C
Source: Front Pediatr. 2022 Jan 21;9:780258. doi: 10.3389/fped.2021.780258 (PMC8815702; doi:10.3389/fped.2021.780258)

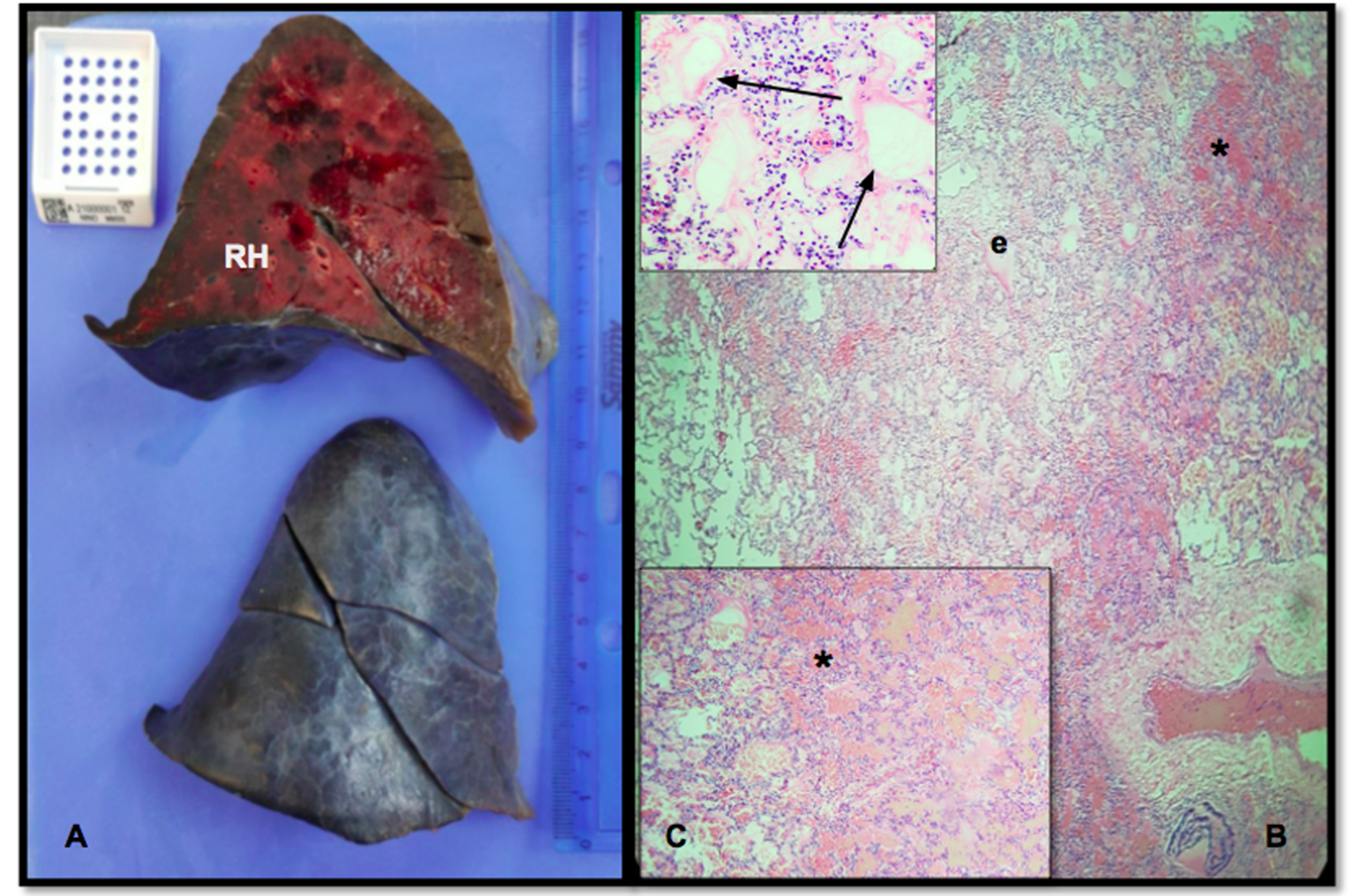

Supplement: Supplementary Figure 1 — Lung. (A) GROSS image: on sectioning, the cut surface is reddish, firm (red hepatization—RH). No mass or gross thrombi are present. (B) ×4 high-power field (HPF) and (C) ×10 HPF—lung parenchyma with edema-e, hyaline membranes (arrow), and hemorrhage coating the alveoli (*). [file Image_1.png]

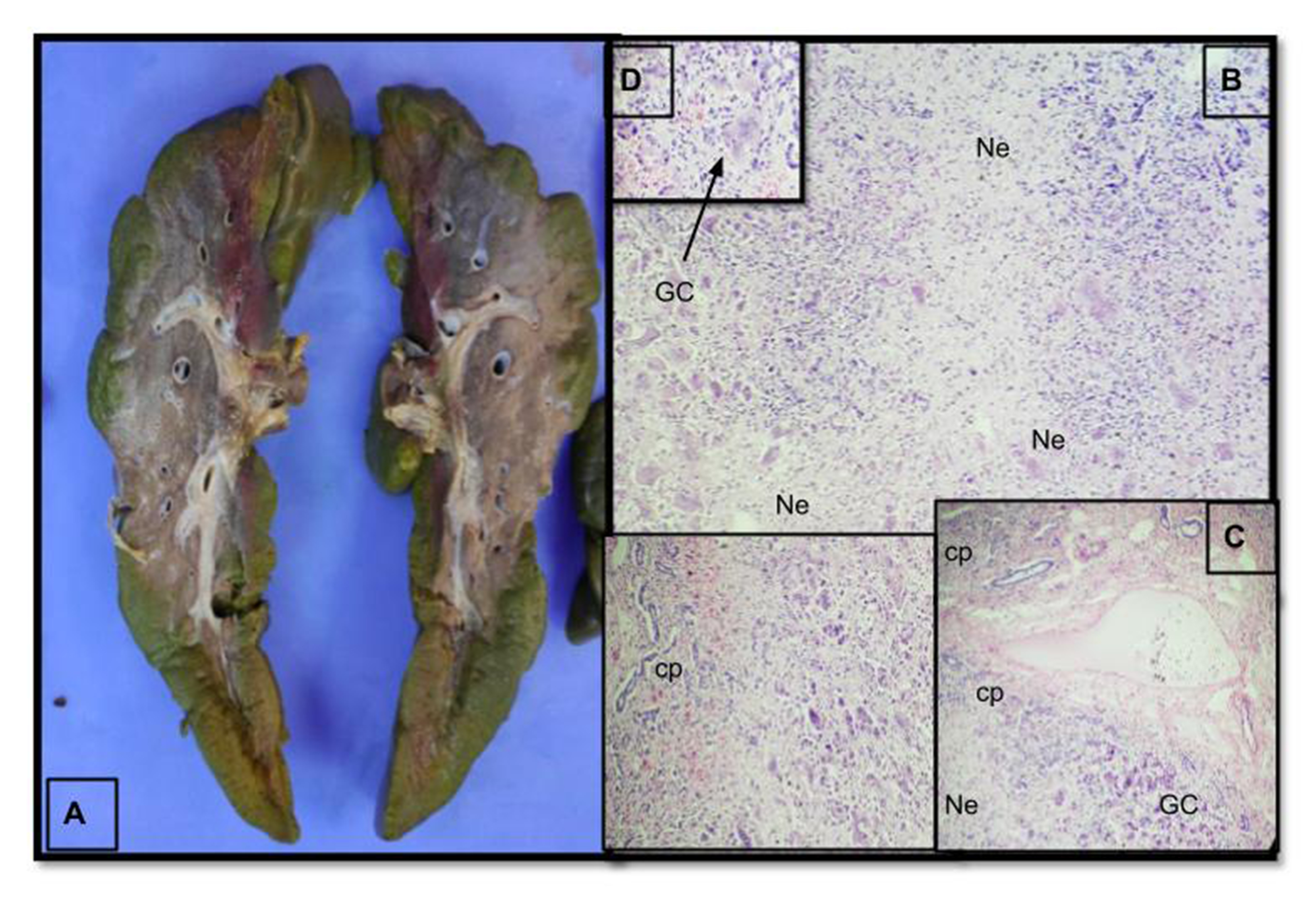

Supplement: Supplementary Figure 2 — (A) GROSS image: The external surface is smooth brown-yellowish, with lobulations. On sectioning, the cut surface is green-yellowish, without masses or other injuries. (B,C) ×10 hpf—massive hepatic necrosis (Ne) (~90%), hepatocyte ballooning with little viable residual parenchyma in zones 2 and 3 (*), observing hepatocytes with multinucleated giant cell transformation (GC), and marked cholangiolar proliferation (cp); (D) ×40 hpf—hepatocytes with multinucleated giant cell transformation (arrow). [file Image_2.jpeg]
